# Health Consumers’ Daily Habit of Internet Banking Use as a Proxy for Understanding Health Information Sharing Behavior: Quasi-Experimental Approach

**DOI:** 10.2196/15585

**Published:** 2020-01-08

**Authors:** Hyeyoung Hah

**Affiliations:** 1 Department of Information Systems and Business Analytics Florida International University Miami, FL United States

**Keywords:** habits, personal health information, information sharing, propensity score, average treatment effect, internet banking, observational data, quasi-experimental design

## Abstract

**Background:**

As the US health care system is embracing data-driven care, personal health information (PHI) has become a valuable resource for various health care stakeholders. In particularly, health consumers are expected to autonomously manage and share PHI with their health care partners. To date, there have been mixed views on the factors influencing individuals’ health data–sharing behaviors.

**Objective:**

This study aimed to identify a key factor to better understand health information sharing behavior from a health consumer’s perspective. We focused on daily settings, wherein health data–sharing behavior becomes a part of individuals’ daily information management activities. Considering the similarity between health and finance information management, we explicitly examined whether health consumers’ daily habit of similar data sharing from the financial domain affects their PHI-sharing behaviors in various scenarios.

**Methods:**

A Web-based survey was administered to US health consumers who have access to and experience in using the internet. We collected individual health consumers’ intention to share PHI under varying contexts, habit of financial information management (operationalized as internet banking [IB] use in this paper), and the demographic information from the cross-sectional Web-based survey. To isolate the effect of daily IB on PHI-sharing behaviors in everyday contexts, propensity score matching was used to estimate the average treatment effect (ATE) and average treatment effect on the treated (ATET) regarding IB use. We balanced the treatment and control groups using caliper matching based on the observed confounding variables (ie, gender, income, health status, and access to primary care provider), all of which resulted in a minimal level of bias between unmatched and matched samples (bias <5%).

**Results:**

A total of 339 responses were obtained from a cross-sectional Web-based survey. The ATET results showed that in terms of sharing contents, those who used IB daily were more likely to share general information (*P*=.01), current information (*P*=.003), and entire data (*P*=.04). Regarding occasions for sharing occasions, IB users were prone to share their information in all cases (*P*=.02). With regard to sharing recipients, daily IB users were more willing to share their personal health data with stakeholders who were not directly involved in their care, such as health administrators (*P*=.05). These results were qualitatively similar to the ATE results.

**Conclusions:**

This study examined whether daily management of similar information (ie, personal financial information) changes health consumers’ PHI-sharing behavior under varying sharing conditions. We demonstrated that daily financial information management can encourage health information sharing to a much broader extent, in several instances, and with many stakeholders. We call for more attention to this unobserved daily habit driven by the use of various nonhealth technologies, all of which can implicitly affect patterns and the extent of individuals’ PHI-sharing behaviors.

## Introduction

As the US health care system is embracing data-driven care, personal health information (PHI) needs to be shared and managed across clinical settings by health consumers [1]. Under this circumstance, PHI, defined as “individually identifiable information relating to the past, present, or future health status of an individual [2],” has become a valuable resource for various health care stakeholders. Recently, the Office of the National Coordinator for Health Information Technology and the Centers for Medicare and Medicaid Services have focused on allowing consumers free and easy access to their health data and enabling them to share PHI even with large technology companies or their selective care counterparts [3]. As health consumers have more choice in care and treatment and electronic access to their structured and unstructured health information [4,5], health consumers are expected to decide the PHI that needs to be shared with their chosen partners and the manner in which it should be shared [6,7]. In other words, individual health consumers should be capable of managing and sharing their PHI across various care contexts.

Health consumers manage not only health information but also nonhealth information on a daily basis. As individuals perform day-to-day functioning in the areas of finance, communication, transportation, socialization, and entertainment [8,9], such activities generate data and require decision-making on accessing, storing, and sharing these data daily. Among these areas, financial information is known to be similar to health information because it is private, personal information that is needed to be shared with strangers (eg, financial advisor), as mandated by government and local policies, and is influenced by individual consumers’ knowledge [10,11], along with its industry-wide movement in consumer-centered services that enables personal financial data to be shared across incumbent banks and nonbanks (ie, Fintech firms) [12]. Thus, individual financial consumers have more power and control over their own data and are expected to make informed decisions. Given that individual consumers can be both health and financial consumers simultaneously, the same individuals’ financial behavior can be a proxy for understanding health information sharing, all of which can provide a useful vantage point for understanding health information–sharing behavior.

Against this backdrop, little attention has been paid to explore health consumers’ health data sharing in the context of daily living. We propose that health consumers are likely to be attracted from similar experiences and may have formed a habit with repeated exposure to the similar tasks while executing health and nonhealth tasks [13]. Taken together, the aim of this paper was to examine health consumers’ existing habits regarding financial information management of the willingness to share health data in various scenarios. More specifically, we focus on three characteristics of health information–sharing behavior with respect to the extent of sharing contents, constituents, and instances [14,15]. By explicitly exploring cross-domain habitual activities in data management, this paper contributes to the ongoing discussion of the expected role of health consumers under the data-driven care system.

## Methods

### Survey Procedure

We recruited participants who were or had the potential to manage and share their own health data electronically. As we particularly focused on individuals who can manage health and financial tasks by the use of relevant technologies, we needed a study sample in which we can capture their daily activities of various data management beyond health care settings. To this end, we contracted with a market research company that had access to the paid panel of health consumers across 50 states in the United States for administering a cross-sectional Web-based survey in 2017. Each participant was incentivized by the completion and quality of their response, which was mainly managed by the market research firm. Our Web-based survey incorporated multiple items that measure health information–sharing behaviors, including health consumers’ intention to share their health information, information sharing contexts, demographic information, and daily technology use such as internet banking (IB) use. An institutional review board approval was obtained before survey distribution. As a result, a total of 339 responses were used for further analysis.

### Survey Instruments

All survey items were sourced from the existing literature, as presented in Table 1. First, for capturing health information–sharing intention with respect to *sharing contents*, *sharing situations*, and *sharing constituents*, we adopted our survey items from the study by Whiddett et al [14] and Anderson and Agarwal [15]. More specifically, sharing contents contained 4 items about the types of information likely to be shared, ranging from general information to all information (including sensitive disease information). *Sharing situations* included four items about the instances in which health consumers are willing to transfer their health information, such as all instances or selective or emergency cases. Finally, *sharing constituents* involved 10 items about whom an individual is willing to share their information with such as physicians, health administrators, and insurance payers. To determine dimensionality of the items for the sharing constituents, we ran exploratory factor analysis (EFA) with varimax rotation using SPSS (IBM Corporation, Armonk, New York). Our EFA identified two factors in *sharing constituents* based on their direct involvement in the care: One dimension is general constituents who do not directly engage in the care (eg, community physicians, government, and health insurance companies), whereas another dimension captures care-related counterparts (eg, physicians, nurses, and pharmacists). The items for health data sharing were anchored on a 5-point Likert scale (1: *strongly disagree* and 5: *strongly agree*). Second, we measured use frequency of daily technology such as computer, internet, email, and IB (ie, “How frequently do you use internet banking?”). These items were anchored on four scales (ie, daily, weekly, monthly, and never), whose anchor was adapted from a mobile banking survey administered by the International Finance Corporation.

Finally, demographic information was captured for gender, marital status, income, education, occupation, race, ethnicity, health condition (chronic disease), and having a primary doctor within the domicile [16].

**Table 1:** Survey items.

Types		Survey items^a, b^	Reference
Sharing contents		General information Current health information Past health information All health information	[14,15]
Sharing instances		In all cases and instances For the purposes of care delivery within the clinical setting For the purposes of other than provision of care (eg, research or marketing) In case of medical emergency conditions	[14,15]
**Sharing constituents**			
	General constituents	Other physicians (who are not involved in your care) at hospitals Other community physicians not involved in your care Health administrators (eg, managers), government agencies Health care researchers Health insurance companies	[14,15]
	Care-related constituents	Physicians (who are involved in your care) at hospitals Other community physicians involved in your care (treating physicians) Nurses Pharmacists	[14,15]
Habitual use of internet banking		Frequency of internet banking use^c^	[17]

^a^We adopted all items from the study by Whiddett et al [14,15].

^b^Information-sharing items are measured on a 5-point Likert scale anchoring on 1 (strongly disagree) to 5 (strongly agree).

^c^Frequency of daily technology use is measured on daily, weekly, and monthly scales adopted from the survey of International Finance Corporation [17].

### Statistical Analysis

The objective of the study was to examine whether frequent use of IB affects when, what, and with whom health consumers are willing to share their own personal health data. However, in observational studies similar to this study, it is often a challenge to isolate the treatment effect because confounding factors can influence both treatment and outcome [18]. To rule out such a confounding effect, prior studies have used various statistical estimation methods such as regression and panel methods, matching estimators, instrumental variables, and regression discontinuity designs [19-21]. Among these methods, we were particularly interested in propensity score matching (PSM), as it has been widely used when randomized clinical trials are infeasible in health care research [20,21]. PSM matches the observed and possible outcomes per each object based on a propensity score—a conditional probability that each observation receives treatment based on a set of observed covariates [22,23]. After matching, average treatment effects (ATEs) can be calculated by averaging out such a difference between the observed and potential outcomes [24]. This method depends on balancing observable covariates in treatment and control groups to isolate and estimate the treatment effect in the presence of confounding effects [25].

In our data, health consumers’ use of IB and health data sharing can be confounded by known factors, that is, demographic characteristics and health status. Following a step-by-step suggestion from the study by Becker and Ichino [26], we conducted PSM (refer to the study by Becker and Ichino [26] for a more detailed explanation), including choice of confounding variables, balancing propensity score and covariates between treatment and control groups, and calculating ATE within the evaluating criteria of bias (the difference between estimated treatment and true effect) and precision of estimated treatment effects. As a first step, it is necessary to choose the correct sets of variables that affect both treatment and outcome variables to better isolate treatment effects. Next, based on the choice of confounding variables, one needs to validate whether there is an overlap in propensity scores between treatment and control groups, which is necessary for drawing an inference by comparing these two groups. After balancing propensity scores in the groups, one needs to check the distribution of covariates within the blocks of propensity score to determine if the treatment and control groups within the block have a similar covariate distribution except for the variation of treatment variable. On the basis of this, the final step is to estimate ATE of focus—either ATE among paired samples within blocks of a propensity score or average treatment effect on the treated (ATET) for the treated observations only [27]. Although there is no clear guideline for calculating sample size for PSM, matching one or two untreated subjects to each treated subject is recommended when using PSM [28].

## Results

### Characteristics of Survey Participants

As shown in Table 2, most of the respondents were working professionals (236/339, 69.6%), female (225/339, 66.4%), and white (269/339, 79.4%). A total of 33.3% (113/339) have chronic conditions, and interestingly, almost all participants believe they are computer literate (337/339, 99.4%). Table 2 presents the demographic characteristics of our survey respondents across three user groups—IB users with daily, weekly, or monthly use frequencies. A majority of respondents use IB weekly (170/339, 50.1%), and participants aged 25-44 years were active users of IB across three user groups (approximately 203/339, 59.9%). We did not find any statistical group difference among demographic characteristics by *t* test (*P*<.05).

Although our data were obtained from a cross-sectional Web-based survey from US health consumers, we further evaluated the representativeness of our sample compared with established benchmark. The Board of Governors of the Federal Reserve System has conducted a Web-based survey on financial consumers’ use of mobile banking in selective years [29]. As we examine the effect of IB use on PHI-sharing behavior, we compared the national profiles of IB consumers from the Federal Reserve Board in 2015 with our sample. We found that age distribution was particularly similar to our sample, as a majority of respondents were aged 25-65 years and a majority of users of mobile banking were aged 25-44 years (Multimedia Appendix 1). Furthermore, we checked age group differences among health consumers with IB frequency using a *t* test and found no significant group differences (*P*<.05).

**Table 2:** Characteristics of survey participants (total number of responses=339).

Demographic variables		All IB^a^ users (N=339), n (%)	Daily IB users (n=96), n (%)	Weekly IB users (n=170), n (%)	Monthly IB users (n=73), n (%)
**Gender^b^**					
	Male	114 (33.6)	34 (35)	54 (31.8)	26 (36)
	Female	225 (66.4)	62 (65)	116 (68.2)	47 (64)
**Marital status**					
	Married	188 (55.5)	51 (53)	103 (60.6)	34 (47)
	Divorced	26 (7.7)	6 (6)	12 (7.1)	8 (11)
	Separated	7 (2.1)	0 (0)	4 (2.4)	3 (4)
	Never married	118 (34.8)	39 (41)	51 (30.0)	28 (39)
**Age (years)**					
	18-24	53 (15.6)	15 (16)	21 (12.4)	17 (23)
	25-34	128 (37.8)	39 (41)	67 (39.4)	22 (30)
	35-44	78 (23.0)	23 (24)	42 (24.7)	13 (18)
	45-54	43 (12.7)	13 (14)	24 (14.1)	6 (8)
	55-64	27 (8.0)	5 (5)	11 (6.5)	11 (15)
	≥65	10 (3.0)	1 (1)	5 (3.0)	4 (6)
**Income status (US $)**					
	<20,000	51 (15.0)	13 (14)	23 (13.5)	15 (21)
	20,000-39,999	76 (22.4)	20 (21)	30 (17.7)	26 (36)
	40,000-59,999	59 (17.4)	22 (23)	28 (16.5)	9 (12)
	60,000-79,999	53 (15.6)	14 (15)	31 (18.2)	8 (11)
	80,000-99,999	44 (13.0)	7 (7)	31 (18.2)	6 (8)
	>100,000	56 (16.5)	20 (21)	27 (15.9)	9 (12)
**Education**					
	Less than high school	9 (2.7)	4 (4)	4 (2.4)	1 (1)
	High school graduate	70 (20.7)	19 (20)	29 (17.1)	22 (30)
	Some college	93 (27.4)	28 (29)	46 (27.1)	19 (26)
	2-year degree	35 (10.3)	11 (12)	14 (8.2)	10 (14)
	4-year degree	85 (25.1)	21 (22)	54 (31.8)	10 (14)
	Master’s degree	40 (11.8)	10 (10)	21 (12.4)	9 (12)
	PhD	7 (2.1)	3 (3)	2 (1.2)	2 (3)
**Occupation**					
	Employed full time	196 (57.8)	65 (68)	102 (60.0)	29 (40)
	Employed part time	40 (1.8)	9 (9)	21 (12.4)	10 (14)
	Unemployed looking for work	29 (8.6)	7 (7)	11 (6.5)	11 (15)
	Unemployed not looking for work	34 (10.0)	10 (10)	15 (8.8)	9 (12)
	Retired	19 (5.6)	1 (1)	9 (5.3)	9 (12)
	Disabled	21 (6.2)	4 (4)	12 (7.1)	5 (7)
**Race**					
	White	269 (79.4)	77 (80)	137 (80.6)	55 (75)
	Black	35 (10.3)	6 (6)	15 (8.8)	14 (19)
	Asian	23 (6.8)	9 (9)	12 (7.1)	2 (3)
	Other	12 (3.5)	4 (4)	6 (3.5)	2 (3)
**Ethnicity**					
	Hispanic	38 (11.2)	10 (10)	21 (12.4)	7 (10)
	Non-Hispanic	301 (88.8)	86 (90)	149 (87.7)	66 (90)
**Chronic conditions**					
	Yes	113 (33.3)	24 (25)	60 (35.3)	29 (40)
	No	226 (66.7)	72 (75)	110 (64.7)	44 (60)
**Primary care access (distance)**					
	Within 5 miles	150 (44.3)	44 (46)	77 (45.3)	29 (40)
	Within 10 miles	129 (38.1)	32 (33)	66 (38.8)	31 (43)
	Within 30 miles	44 (13.0)	15 (16)	20 (11.8)	9 (12)
	Not available	16 (4.7)	5 (5)	7 (4.2)	4 (6)

^a^IB: internet banking.

^b^n=236 for all IB users.

### Propensity Score Matching Results

**Figure 1:**
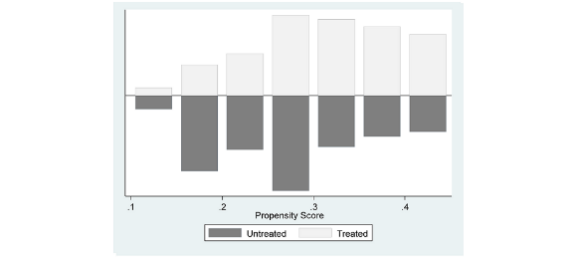
Distribution of propensity score between treated and untreated groups.

We conducted PSM analysis using Stata version 14.2 software (College Station, Texas). In our analysis, we chose income, race, health status, and having a primary care doctor within close proximity as our confounding variables, among others, which are likely to influence both IB use and health information sharing behavior. Subsequently, the propensity score was calculated for each block using a logit model. Figure 1 presents the distribution of propensity scores between the treated and untreated groups within blocks, termed as common support (propensity score overlaps in the matched pairs for each block), with appropriate overlap across blocks of propensity scores [30].

**Figure 2:**
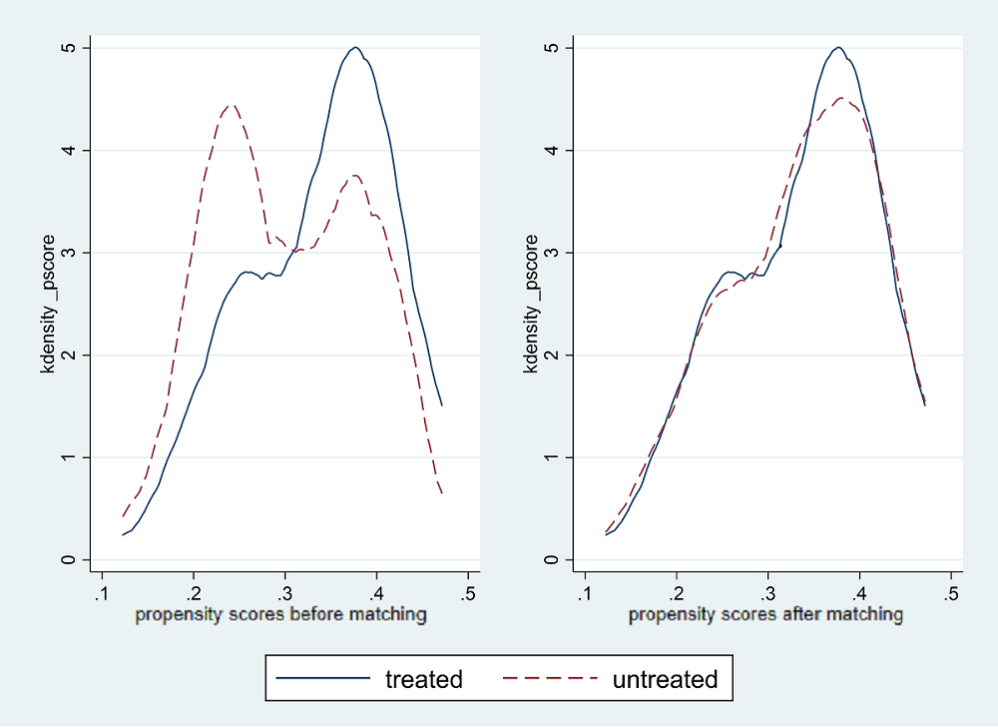
Density plots in health information sharing behavior. Treated sample comprised daily users of internet banking, and the rest of the users were included in the untreated group.

Table 3 illustrates covariate balancing among the variables before and after matching. It indicates that standard bias (percentage of bias) was less than 5% after caliper matching, and it is reasonable to move forward with the next step of analysis. To match a treated individual with nontreated individuals using similar propensity score, we used caliper matching (0.2×standard deviation of logit of propensity score with 1:2 neighbor matching with replacement) [26]. Figure 2 demonstrates a similar distribution of information-sharing behavior (sharing content) at baseline before and after caliper matching. Thus, we proceeded with ATE and ATET.

**Table 3:** Covariate balance before and after propensity score matching.

Variables		Unmatched sample					Matched sample			
		Mean, Treated	Mean, Untreated	Bias (%)	*P* value	Mean, Treated		Mean, Untreated	Bias (%)	*P* value
**Gender**										
	Female	0.65	0.67	−5	.69	0.65		0.65	0	>.99
**Income**										
	20,000-39,999	0.22	0.23	−1.6	.90	0.22		0.22	0	>.99
	40,000-59,999	0.21	0.15	15.4	.21	0.21		0.21	0	>.99
	60,000-79,999	0.16	0.16	1.1	.93	0.16		0.16	0	>.99
	80,000-99,999	0.08	0.15	−21.8	.10	0.08		0.08	0	>.99
	>100,000	0.22	0.15	19.4	.11	0.22		0.22	0	>.99
**Chronic condition**										
	No	0.72	0.63	17.9	.16	0.72		0.72	0	>.99
**Primary care access (distance)**										
	Within 5 miles	0.42	0.44	−2.6	.84	0.42		0.42	0	>.99

### Average Treatment Effects of Daily Internet Banking Use

Finally, we estimated ATE and ATET as displayed in Tables 4 and 5, respectively. Here, ATE describes the ATE of IB use on health information sharing by comparing the treated (daily use of IB) and untreated groups (nondaily use of IB) in the matched pair sample. ATET indicates the estimated average effect of daily use of IB on health information sharing among daily IB users. We defined a treatment effect of IB as the use of IB on a daily basis, as we viewed daily use of IB as health consumers’ daily habit. Prior literature noted that habit is an automatic response to be formed by more frequent use and exposure of a focal event, technologies, and tasks [31]. Thus, we can consider daily use of IB as an existing habit of managing private personal information compared with weekly or monthly use of IB in our data. As such, we proceed to examine the treatment effect of daily use of IB on health information sharing under various circumstances.

In Table 4, there are differential behaviors in sharing contents, situations, and constituents between the treated and untreated groups. Daily IB users were more willing to share general and current health information (*P*=.009 and *P*=.004, respectively) for all situations (*P*=.06). For sharing constituents, these individuals were prone to share their information with non–care-related personnel, such as health administrators at a hospital (*P*=.05). Table 5 presents the average effect of daily IB use on health information sharing among daily IB users. We found that the treated individuals were likely to share all PHI with sensitive contents (*P*=.04) for all situations (*P*=.02). Moreover, they shared more PHI with non–care-related health administrators within the hospital (*P*=.05). Overall, results from ATE and ATET were qualitatively similar.

**Table 4:** Average treatment effect of daily internet banking use for matched pair sample.

Outcomes				Coefficient		SE		Z score		*P* value		95% CI	
**Sharing contents**													
		General information		0.324		0.124		2.61		.009		0.081 to 0.567	
		Current information		0.364		0.125		2.91		.004		0.119 to 0.61	
		Past information		0.17		0.127		1.34		.18		−0.08 to 0.42	
		Full information		0.215		0.153		1.4		.16		−0.085 to 0.514	
**Sharing instances**													
		All cases and situations		0.281		0.151		1.87		.06		−0.014 to 0.577	
		Care purposes		0.131		0.111		1.18		.24		−0.086 to 0.349	
		Noncare purposes		0.086		0.178		0.48		.63		−0.263 to 0.435	
		Medical emergency		0.133		0.095		1.39		.16		−0.054 to 0.32	
**Sharing constituents**													
	**For direct care**												
			Your physician		0.084		0.114		0.74		.46		−0.139 to 0.307
			Involving community physician		−0.064		0.122		−0.53		.60		−0.304 to 0.175
			Nurses		0.119		0.139		0.85		.39		−0.154 to 0.393
			Pharmacists		−0.023		0.174		−0.13		.89		−0.364 to 0.317
	**For indirect care**												
			Noninvolving physician at hospital		0.278		0.191		1.45		.15		−0.097 to 0.653
			Noninvolving community physician		0.234		0.187		1.25		.21		−0.132 to 0.601
			Health administrators (eg, managers)		0.347		0.174		1.99		.05		0.005 to 0.688
			Government		0.23		0.189		1.22		.22		−0.14 to 0.601
			Health care researchers		0.179		0.185		0.96		.34		−0.185 to 0.542
			Insurance		0.169		0.179		0.94		.35		−0.183 to 0.521

**Table 5:** Average treatment effects on the treated of daily internet banking use.

Outcomes				Coefficient		SE		Z score		*P* value		95% CI	
**Sharing contents**													
		General information		0.346		0.140		2.470		.01		0.071 to 0.621	
		Current information		0.399		0.134		2.960		.003		0.135 to 0.662	
		Past information		0.208		0.145		1.430		.15		−0.076 to 0.492	
		Full information		0.334		0.160		2.090		.04		0.021 to 0.647	
**Sharing instances**													
		All cases and situations		0.319		0.139		2.300		.02		0.047 to 0.591	
		Care purposes		0.192		0.117		1.640		.10		−0.037 to 0.421	
		Noncare purposes		0.156		0.200		0.780		.44		−0.236 to 0.547	
		Medical emergency		0.179		0.104		1.710		.09		−0.026 to 0.383	
**Sharing constituents**													
	**For direct care**												
			Your physician		0.058		0.136		0.430		.67		−0.208 to 0.324
			Involving community physician		−0.119		0.139		−0.860		.39		−0.392 to 0.153
			Nurses		0.184		0.139		1.320		.19		−0.089 to 0.457
			Pharmacists		0.021		0.155		0.140		.89		−0.283 to 0.326
	**For indirect care**												
			Noninvolving physician at hospital		0.331		0.200		1.660		.10		−0.060 to 0.722
			Noninvolving community physician		0.201		0.199		1.010		.31		−0.188 to 0.590
			Health administrators (eg, managers)		0.350		0.177		1.980		.05		0.003 to 0.698
			Government		0.232		0.168		1.380		.17		−0.097 to 0.561
			Health care researchers		0.146		0.177		0.820		.41		−0.202 to 0.493
			Insurance		0.249		0.152		1.640		.10		−0.049 to 0.548

## Discussion

### Principal Findings

Despite growing expectation of individuals’ responsibility for sharing PHI for data-driven care, there have been mixed results regarding factors influencing health consumers’ sharing intention under various circumstances. Given that management of financial information resembles that of health information, this study called for more attention on individuals’ daily use of financial information management as a proxy for health information sharing and hypothesized that the more frequently individuals managed their financial information, the more likely they were willing to share PHI under various sharing conditions. Our PSM results revealed that daily IB users were more willing to share the large extent of health information for all instances, even with personnel who were not directly involved in their care process. This is one of the first studies that explores the role of a daily habit of financial information management to predict individuals’ intention in sharing their health information.

### Limitations

Although we presented important findings on the role of daily habit of IB use, our results should be interpreted with caution because of their limitations. First, our study is conducted via a cross-sectional Web-based survey. Although our research question was aligned with our study design, tracking health consumers’ information management behavior over time can provide an in-depth view and further identify contextual factors in the daily living context. The cross-section time series information on health consumers’ information management in daily living would be beneficial in the future to capture granular level of measures and to control unobserved heterogeneity among individuals. Second, we conceptualized our treatment effect as habitual use of financial data management and operationalized it by the daily use of IB. Although our unidimensional, binary measure of IB use was appropriate for PSM methodology, future research can incorporate multi-item measures to capture multidimensional aspects of financial data management for health consumers in the richer research models. Finally, we acknowledge that majority of survey respondents in our study have no immediate health issues (237/339, 69.9%); therefore, the results of this study may not be generalized to patients who have various health conditions and statuses. As we assumed that the same individuals can be both health and financial consumers, the findings of this study can be a baseline information to compare individuals’ behaviors in medical situations in subsequent research.

### Comparison With Prior Work

#### Role of the Existing Habit in Daily Living

In this paper, we first examined the daily habit that has not been of focus in health care research. More specifically, this paper juxtaposed the similarity between health and financial data and identified financial data habit as a key factor in understanding health data sharing from the same individuals. Our approach assumed that the same individuals are customers for both health and financial services; therefore, such individual-level behaviors can be closely related. Theoretically, it is known that when people cope with a new event, their reaction might be predictable simply because there are likely to base their reaction on past experience or their knowledge of similar situations [32,33]. Defined as an automatic reaction toward certain stimuli or inputs based on past experience or learning [34,35], habit has been a key research variable to predict a certain behavior in education, health care, and information systems disciplines [10,36]. In the information search context, people seek information from easily available internet sources or acquaintances (eg, friends and family), and they typically return to such habits for future information-seeking behavior [33]. In technology use context, prior learning and habits from technology can influence adoption and use of a new technology [37,38]. Another example noted that health consumers’ health status and Web-based community membership (ie, PatientsLikeMe) makes them feel comfortable sharing their sensitive health information with people who are not directly related to their care [39]. Thus, an individual’s habitual behavior in one life area can affect the same individual’s behavior in another life setting.

Although prior health care literature highlighted the importance of individuals’ habit to understand health behaviors, habit has been mainly defined within the context of health care. For example, health consumers’ exposure to personal health records technology influenced individuals’ share of PHI with care providers and non–care-related providers [35,40]. Given the complexity of PHI and heterogeneous information sharing behaviors, there is a growing interest in understanding health consumers’ differential information sharing. Previous seminal papers have documented that sharing of the most sensitive PHI varies widely under various conditions, with sharing counterparts—care-involved personnel (eg, family and caregivers) or a broad audience (government or non–care-related providers) [41-43]. Although prior studies point out to their existing predisposition on data security [44], familiarity with technology use [35,43] or Web-based community membership [40] as plausible reasons for such heterogeneous responses, these factors have been searched and identified in clinical settings. By recontextualizing health data sharing into everyday living contexts, we explicitly examined whether decisions on health information sharing are influenced by daily, nonhealth-related habit of individual consumers.

#### Lesson Learned From the Similarity Between Health and Financial Data

This paper also showed that frequent exposure to IB is positively related to health data–sharing behavior. This finding is in line with and extends prior health research in two ways. First, although the effect of internet use has been widely discussed to understand health information sharing behavior, the influence of habitual internet use is lesser known [40]. At a granular level, we identified that daily users of IB are more prone to share more data for all cases and with non–care-related stakeholders. Second, this study identified opportunities to identify new phenomena from financial activities of individual health consumers. In the financial sector, individual consumers have more power and control over their own data and are expected to make informed decisions in various situations under the nationwide trends of consumer-driven service. A recent survey shows that 60% of consumers are willing to share personal data (eg, location data and lifestyle information) with financial service providers in exchange for customized promotion and better services, and young tech-savvy customers are willing to share more data [45]. Yet, such data sharing is based on each consumer’s autonomous decision about sharing across traditional banks and nonbanks (ie, Fintechs) [46]. Viewing health information sharing as a part of multidimensional information managing tasks in everyday living, this study clearly demonstrated that a cross-domain habit of information management is positively associated with health information sharing, which has largely been underexplored in health care research [47].

### Future Research

For future research, it will be worthwhile to revisit this research model in clinical setting and explore the effect of habit on health data sharing in the clinical setting for those who have chronic conditions or medical urgency. Health data sharing is a complex and variable phenomenon, and more interdisciplinary research is indispensable. As health consumers are attaining more ownership to manage and share their own health information via websites, wearables, and mobile apps, it is important to determine whether they are capable of dealing with this volume of data [48] and whether they make informed decisions to share personal data with multiple stakeholders. Various types of structured and unstructured health information may be stored at an individual’s home or workplace or at a hospital. Given that such data need to be freed by the hands of health consumers and distributed for the optimal health care decision and outcomes [49], future research can further explore various information type and their willingness to share such data under different sharing scenarios.

### Practical Implications

This research has practical implications. First, health technology vendors may design health information management tools modeled after financial information management tools [50]. As health care and finance are both characterized by the important role of system usability for customer satisfaction, involving more consumers in the rapid process of innovation and understanding consumer behaviors and fast-moving information technology trends [51], system design can support health consumers’ willingness to share their own data and further liberate their data for consumer-centered care [52]. To better manage health information for self-health management [53], individual health consumers need to collectively as well as selectively manage either type of health information and use it for their own medical conditions and contexts. Health technology can support such sharing behavior by providing repository of health data that is similar to a consolidated bank account and allowing them to selectively share or store it [51]. Second, health consumers need to be educated on how to manage various types of health information, including access, process, and exchange of public and private health information. As health information is domain-specific and either personal [15] or widely available on the internet [54], individual health consumers should understand the difference between public and private health information and shareability of such information accordingly. As shown in our results, even with the experience of managing sensitive financial data daily, health consumers may not be ready to share PHI with other stakeholders who are not directly involved in their care. This has timely implications on the direction and content of health consumer education about sharing constituents [55].

### Conclusions

The objective of this study was to examine the effect of daily use of financial technology on health information sharing. Considering the similarity between health and financial technology and the characteristics of such information, this study proposes that the unobserved habit of managing sensitive information daily can further affect managing and sharing another type of sensitive information—PHI. Results from PSM reveal that frequent users of financial technology are more prone to share their entire health information in all instances, even with non–care-related stakeholders. Subsequent research can explore more granular types of habits in various life domains to better understand health consumers’ readiness to manage self-health information for realizing consumer-centered care in the future.

## Appendix

Multimedia Appendix 1 National profiles of mobile banking users.

## Abbreviations

ATE: average treatment effect
ATET: average treatment effect on the treated
EFA: exploratory factor analysis
IB: internet banking
PHI: personal health information
PSM: propensity score matching
