# Supplementary material for: Health Consumers’ Daily Habit of Internet Banking Use as a Proxy for Understanding Health Information Sharing Behavior: Quasi-Experimental Approach
Source: J Med Internet Res. 2020 Jan 8;22(1):e15585. doi: 10.2196/15585 (PMC6996727; doi:10.2196/15585)
Supplement: Multimedia Appendix 1 [file jmir_v22i1e15585_app1.docx]

### Multimedia Appendix A

Table 1. National profiles of mobile banking users ^a^

|  | Frequency | % |
| --- | --- | --- |
|  |  |  |
| Age |  |  |
| 18-24 | 73 | 11.40 |
| 25-34 | 182 | 28.40 |
| 35-44 | 129 | 20.20 |
| 45-54 | 113 | 17.70 |
| 55-64 | 99 | 15.50 |
| 65-74 | 36 | 5.60 |
| 75+ | 8 | 1.30 |
| Total | 640 | 100 |

^a^ This sample was obtained from Federal Reserve System’s consumer financial survey in 2015.
